# Defining saliva and tooth biofilm microbiota in adolescents in a low caries community and characterization by caries status

**DOI:** 10.1080/20002297.2017.1325193

**Published:** 2017-05-30

**Authors:** Linda Eriksson, Pernilla Lif Holgerson, Ingegerd Johansson

**Affiliations:** ^a^ Umeå University

## Abstract

The present study evaluated the microbiota of saliva and tooth biofilm by sequencing segments of the 16S rRNA gene using the common Illumina MiSeq (v3–v4) and the newer PacBio (v1–v8) sequencing platforms and searched for associations with caries in a low-caries population with regular dental care since early childhood. Saliva and tooth biofilm from adolescents and mock bacteria communities were analysed, including validity and reliability estimates. Caries was scored at baseline and 2 years later. The two sequencing platforms revealed similar microbiota patterns for saliva and tooth biofilm, respectively. Saliva microbiota discriminated caries-affected adolescents from caries-free adolescents with enumeration of five species in caries-affected participants and one species thereof predicting 2-year caries increment. Reliability was high and validity was acceptable if appropriate inclusion criteria were determined.

